# Uphill diffusion and overshooting in the adsorption of binary mixtures in nanoporous solids

**DOI:** 10.1038/ncomms8697

**Published:** 2015-07-16

**Authors:** Alexander Lauerer, Tomas Binder, Christian Chmelik, Erich Miersemann, Jürgen Haase, Douglas M. Ruthven, Jörg Kärger

**Affiliations:** 1 Faculty of Physics and Earth Sciences, University of Leipzig, Linnéstraße 5, 04103 Leipzig, Germany.; 2 Department of Mathematics, University of Leipzig, Augustusplatz 10/11, 04109 Leipzig, Germany.; 3 Department of Chemical and Biological Engineering, University of Maine, 5737 Jenness Hall, Orono, Maine 04469, USA.

## Abstract

Under certain conditions, during binary mixture adsorption in nanoporous hosts, the concentration of one component may temporarily exceed its equilibrium value. This implies that, in contrast to Fick's Law, molecules must diffuse in the direction of increasing rather than decreasing concentration. Although this phenomenon of ‘overshooting' has been observed previously, it is only recently, using microimaging techniques, that diffusive fluxes in the interior of nanoporous materials have become accessible to direct observation. Here we report the application of interference microscopy to monitor ‘uphill' fluxes, covering the entire period of overshooting from initiation until final equilibration. It is shown that the evolution of the profiles can be adequately predicted from the single-component diffusivities together with the binary adsorption equilibrium data. The guest molecules studied (carbon dioxide, ethane and propene) and the host material (ZSM-58 or DDR) are of practical interest in relation to the development of kinetically selective adsorption separation processes.

## Abstract (web summary)

During the transient adsorption of a binary mixture in a nanoporous host, the concentration of one component may temporarily exceed its equilibrium value, with molecules diffusing in the direction of increasing concentration. Here, the authors use microimaging to examine this process in a real system.

## Graphical abstract

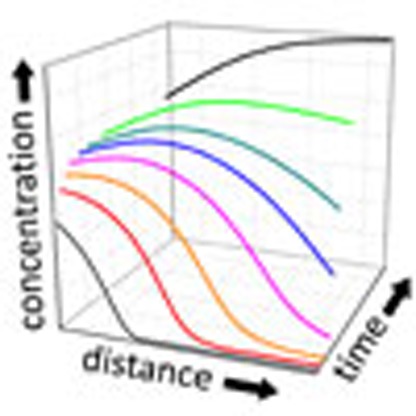

Atoms and molecules are, as a consequence of their thermal energy, subject to permanent random movement (Brownian motion). This process, referred to as diffusion, is a universal phenomenon that occurs, on different timescales, in all states of matter^1^,^2^. It is critically important in many catalytic and separation processes involving the application of nanoporous materials^3^,^4^,^5^,^6^,^7^. In these processes, the separation is achieved based on differences in the flux rates of the different components resulting from differences in their adsorption equilibria and intracrystalline diffusivities. Given the differences in molecular uptake, during multi-component adsorption, different components are easily understood to penetrate into the nanoporous host materials at different rates. These differences are exploited in the ‘kinetic' mode of mass separation^8^.

During the initial stage of the uptake, the more mobile species diffuses rapidly into the interior of the nanoporous crystal, approaching a concentration dictated by the single-component equilibrium isotherm. With continuing molecular uptake, the slower component will eventually diffuse throughout the crystal and the concentrations of both components will approach uniform equilibrium levels determined by the binary isotherm. However, during this equilibration process, the slower diffusing molecules must compete with the already accommodated faster molecules, leading to a decrease in the local concentration of the latter. Consequently, during uptake, the concentration of the faster component ‘overshoots', exceeding its final equilibrium value. Hitherto experimental studies of this phenomenon were necessarily based only on measurements of the time-dependent average concentrations of both components in the adsorbed phase^9^,^10^,^11^. It was not possible to observe the evolution of the intracrystalline concentration profiles directly or to explore the elementary transport processes responsible for this effect.

It was only with the introduction of infrared (IR) imaging^12^,^13^,^14^,^15^ that it became possible to record simultaneously the evolution of the concentration profiles of different guest molecules in a nanoporous host. However, owing to limitations inherent to IR spectroscopy, the minimum spatial resolution in such studies is about 2 μm (refs 12, 13). Substantially higher resolution (<1 μm) can be achieved using interference microscopy (IFM)^12^,^14^,^16^,^17^,^18^,^19^. Previous studies by this technique have revealed the existence of both surface barriers and intracrystalline transport barriers as well as preferential diffusion through different pore systems in zeolites such as ferrierite^14^. However, these studies have so far been limited to the observation of single-component adsorption. In this paper, we show that, under favourable conditions, IFM can also be applied to record the transient profiles of the individual species during multi-component adsorption, with a spatial resolution of <1 μm. The systems studied (mixtures of CO_2_–C_2_H_6_ (refs 20, 21, 22) and C_2_H_6_–C_3_H_6_ (refs 23, 24, 25) in zeolite ZSM-58, also known as DDR^26^,^27^) turn out to be particularly advantageous, giving rise to special situations in which IFM is able to provide detailed information about the local concentrations of the individual components rather than just total concentrations. These options are based on the large difference between the diffusivities of the two components considered. For CO_2_–C_2_H_6_ mixtures, the diffusivity of the faster component (CO_2_) is high enough so that, on the timescale of our measurements, the local CO_2_ concentration attains its equilibrium value (determined by the external CO_2_ pressure and the local C_2_H_6_ concentration) essentially instantaneously. With C_2_H_6_–C_3_H_6_ mixtures, vice versa, the diffusivity of the slower component (C_3_H_6_) is small enough so that C_2_H_6_ (now the faster component!) equilibrates, with the C_3_H_6_ profiles remaining essentially unchanged. Finally, it is shown that the evolution of the concentration profiles, including both ‘overshooting' and final equilibration, can be predicted from the binary adsorption equilibrium isotherm and the single-component diffusivities.

## Results

### Recording single components during mixture adsorption

By using characteristic frequencies of absorption as a ‘finger print' for a given molecular species, microimaging by IR microscopy is able to distinguish between different molecular species and adsorption sites^28^,^29^,^30^. Owing to this ability, IR microscopy has been recognized as a powerful tool for investigating the spatial-temporal dependence of guest concentrations in nanoporous materials during both multi-component adsorption^7^,^14^,^31^,^32^,^33^,^34^ and catalytic reactions^12^,^15^. This capability, however, comes at the cost of spatial resolution which, even under favourable conditions, is limited to a couple of micrometres. IFM, by contrast, is able to attain spatial resolutions of 0.45 × 0.45 μm^2^ within the plane of observation. However, in most situations IFM can only provide information about overall loadings rather than the concentrations of individual components. In the present study, we succeeded in identifying and exploiting two special cases in which these limitations can be circumvented, making it possible to take advantage of the superior spatial resolution of IFM to study in detail the transient behaviour of a two-component adsorption system.

**Figure 1:**
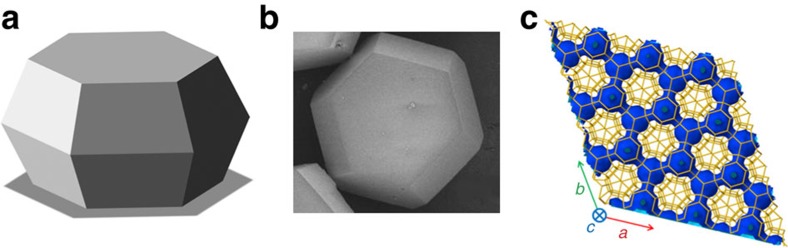
Host system under study. (**a**) Graphic representation of a single crystal of type ZSM-58 (ZSM=Zeolite Socony Mobil). An electron micrograph is presented in **b**, revealing the hexagonal prism shaped crystal habit of these zeolites (diameter of about 34 μm). (**c**) shows the framework of ZSM-58 (yellow sticks) as well as the pore structure (surface coloured in blue), referred to as structure type DDR (Deca-dodecasil 3R)^56^. Cages and windows have diameters of 0.77 nm and 0.36 to 0.44 nm, forming a hexagonally symmetric two-dimensional pore system. In both **b** and **c**, the *c* axis is aligned perpendicular to the plane of the paper (*a*–*b* plane). Mass transport occurs exclusively in this plane as the window size in the *c* direction (0.26 nm) is too narrow for guest molecules to pass through.

The application of IFM for microimaging is based on the fact that changes in guest concentration give rise to changes in the refractive index and therefore to changes in the phase shift between a light beam passing the crystal and a reference beam passing through the surroundings. To a first-order approximation, changes in the phase shift are related linearly to the concentration changes^12^,^14^:


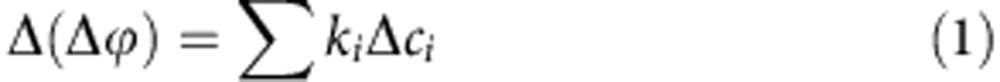


with Δ*c*_*i*_ denoting the change in the concentration of component *i*. Equation (1) is strictly valid only if there are no diffusion fluxes in the observation direction. This should be true for ZSM-58 since the pore system is two-dimensional with no channels in the direction of the crystal axis. The structure is shown in Fig. 1. *k*_*i*_ stands for the proportionality factor between changes in the concentration of component *i* and phase changes and may be determined by comparison with the results of conventional adsorption measurements. It is evident that, even for a two-component system with known parameters *k*_*i*_ (*i* =1, 2), experimental measurements of changes Δ(Δ*ϕ*) in the phase shift (the primary parameters of IFM) do not provide sufficient information to determine the changes Δ*c*_1_ and 
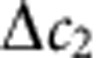
 for the individual components. However, such a separate determination may become possible if the difference in the equilibration rates of the two components is sufficiently large. The information actually accessible depends on the relation between these equilibration rates and the timescale of the IFM measurements, which is determined by the temporal resolution (currently about 10 s (ref. 14)).

In one limiting case, realized in the present study for the uptake of CO_2_–ethane by zeolite ZSM-58, the diffusion rate of one component (CO_2_) is fast enough to ensure essentially instantaneous equilibration of this component over the sample, with the local concentration therefore determined entirely by the partial pressure of this component in the surrounding atmosphere and the local concentration of the second component (in this case, ethane). Under such conditions, equation (1) may be rewritten as


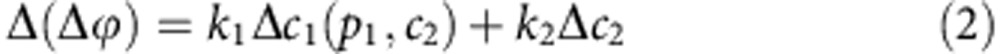


where the indices 1 and 2 refer to the fast (CO_2_) and slow (ethane) components, respectively. Since, by assuming local equilibration of the fast component, the concentration *c*_1_ and, hence, its variation Δ*c*_1_ are known functions of *p*_1_ and *c*_2_, Δ*c*_2_ becomes the only unknown in equation (2).

**Figure 2:**
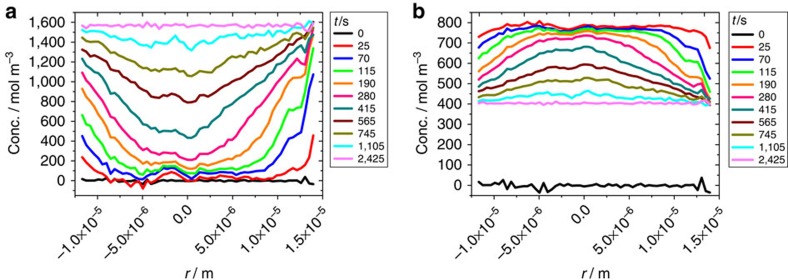
Single-component profiles during uptake of C_2_H_6_–CO_2_ mixtures. (**a**) Evolution with time of the concentration profiles for ethane (the slow component), after the initially activated crystal of zeolite ZSM-58 has been brought into contact with a C_2_H_6_–CO_2_ mixture with partial pressures of each component of 200 mbar in the surrounding atmosphere. With the ethane front penetrating the system, CO_2_ is partially desorbed as shown in **b**. Finally both components reach uniform equilibrium concentrations determined by their partial pressures.

Figure 2 shows a summary of the transient concentration profiles determined in this way for ethane (Fig. 2a) and CO_2_ (Fig. 2b) during molecular uptake, induced by bringing an initially activated crystal of zeolite ZSM-58 into contact with an atmosphere with partial pressures of about 200 mbar for ethane and 200 mbar for CO_2_. In the Methods section (see also Supplementary Figs 1–3), we describe in further detail how these profiles have been determined from the primary IFM data by incorporating the information from the two-component adsorption isotherm as provided by the ideal adsorbed solution theory (IAST)^35^. For the system under study, IAST has been found to provide an accurate prediction of the binary equilibrium over the relevant pressure range^36^. We will return to a more detailed discussion of the evolution of the transient concentration profiles at the end of the next subsection where the initial period of uphill diffusion and overshooting will be shown to be followed by final equilibration.

**Figure 3:**
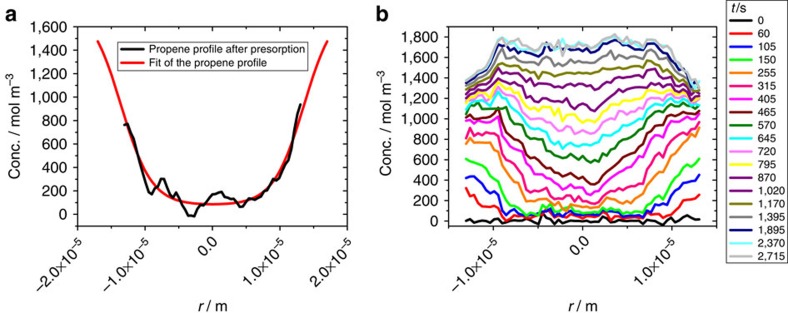
Single-component profiles during C_2_H_6_ uptake with presorbed propene. (**a**) Distribution of propene within the crystal of zeolite ZSM-58 after nearly 7 h of presorption at a pressure of 10 mbar in the surrounding atmosphere, directly before a partial pressure of 200 mbar of ethane was introduced to the system. The measured profile has been approximated by a smoothed curve. (**b**) Evolution of the ethane concentration with time in the presence of the constant (presorbed) concentration of propene.

A second possibility that allows the evolution of the concentration of a particular component to be followed by IFM (during multi-component adsorption) is the other limiting case in which the uptake of one component is slow enough to be followed by IFM but still sufficiently fast that the concentration profiles of the other component may be assumed to remain essentially invariant during uptake of the fast component. For this type of experiment we have, once again, used ethane, now as the fast component, in a mixture with propene as the slow component (see Methods and Supplementary Figs 4 and 5). With equilibration times of several days, the propene concentration may in fact be considered to remain essentially constant during the uptake of ethane. As an example, Fig. 3b shows the evolution of the ethane concentration in a crystal of zeolite ZSM-58, induced by a pressure step from 0 to 200 mbar of ethane in the surrounding atmosphere. Before exposure to ethane, the zeolite crystal was exposed to a propene atmosphere of 10 mbar over nearly 7 h, after which the intracrystalline concentration profiles of propene had attained the form shown in Fig. 3a.

### From uphill diffusion to final equilibration

The onset of ethane uptake as revealed by the concentration profiles shown in Fig. 3b follows the common pattern. Although guest concentrations at the boundary itself are inaccessible to direct observation as a consequence of the beveled side faces of the crystals (see Fig. 1b), ethane concentrations are seen to increase rapidly in the vicinity of the crystal boundary and to decay towards the crystal centre. The ethane concentration within the crystal increases and the fluxes, giving rise to this increase, are seen to be directed ‘downhill' during this period of time, that is, in the direction of decreasing concentration. About 720 s after the introduction of ethane, the ethane concentration within the crystal reaches the concentration level close to the surface. Remarkably, however, the ethane concentration within the crystal continues to increase further, with concentrations considerably exceeding the concentrations near the crystal surface: the diffusive flux of the ethane molecules is thus seen to continue to be directed towards the crystal interior, even though this is now the direction of increasing ethane concentration. In other words, we now see diffusion ‘uphill'.

To understand this observation, we have to recognize that diffusive fluxes are driven by the gradients of the chemical potentials. In a binary adsorbed phase, the chemical potential of each species depends on the concentrations of both components, with the result that the gradient of the chemical potential may be represented as a linear combination of the concentration gradients of the different molecular species within the system, resulting in Fick's generalized first law^2^,^37^,^38^,^39^,^40^


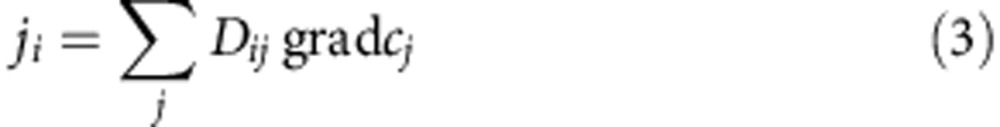


with the generalized diffusivities (elements *D*_*ij*_ of the diffusion matrix) correlating the flux of species *i* with the concentration gradient of species *j*. Uphill diffusion, that is, diffusive fluxes (in this case of ethane) in the direction of increasing concentration, may thus be easily explained by the presence of a second component (propene) with a concentration profile that decreases sufficiently rapidly in the direction of the ethane flux.

Provided that there is no significant barrier at the crystal surface, the ethane concentration close to the surface will assume its equilibrium value as determined by the gas phase composition. With concentrations in the crystal interior exceeding the boundary concentration, the overall concentration of ethane is seen to exceed the equilibrium value as determined by the partial pressures of the constituents of the surrounding gas phase. This phenomenon of ‘overshooting' in the amount adsorbed has been predicted theoretically^9^,^10^,^11^,^41^,^42^,^43^ and observed experimentally in uptake measurements^7^,^9^,^11^,^43^. With the potential of microimaging, the exploration of overshooting phenomena may now be based on the *in situ* observation of the evolution of the guest profiles.

Within the timescale of ethane uptake as considered in Fig. 3b, the overshoot reaches its maximum after about 2,300 s. Changes in the concentration profiles recorded in the remaining period of about 400 s of the total observation of 2,715 s, as realized in these studies, are negligibly small. As a prerequisite for this stationary behaviour, the ethane flux must vanish during this period. With equation (3), the ethane fluxes are seen to vanish when, throughout the crystal, the gradients of the ethane concentration have attained a certain, well-defined maximum value, determined by the gradient of the propene concentration at this same position and the two relevant elements of the diffusion matrix (which predict the ethane flux from the two concentration gradients).

To continue the observation of overshooting, one would have to switch to a much longer timescale (large enough to cover the uptake or release of propene). Within this timescale, the concentration of ethane molecules would assume, essentially instantaneously, their equilibrium values determined by the local propene concentrations and the ethane pressure in the surrounding atmosphere. Overshooting terminates with the formation of a homogeneous propene distribution at which point the ethane molecules are also distributed homogeneously throughout the crystal, with both concentrations determined by their partial pressures in the surrounding atmosphere.

With uptake and release times of several days (Fig. 3a and Supplementary Fig. 5, see Methods), the propene diffusivity in zeolite ZSM-58 is much too low to allow such experiments to be carried out within a reasonable time frame. Experiments of exactly this type are however possible with the first system considered in this study, that is, with ethane as the ‘slow' molecule in a mixture with CO_2_.

To continue the discussion of the process of overshooting, we return to Fig. 2 that shows the evolution of the transient concentration profiles of ethane and CO_2_ following the exposure of the (initially empty) crystals to an atmosphere consisting of a mixture of these two species. CO_2_ is now the ‘fast' component (taking on the role of ethane in the experiments shown in Fig. 3). Its concentration at all points within the crystal may therefore be assumed to reach its equilibrium value, as determined by the external CO_2_ pressure and the ethane concentration at that position. Owing to the very high CO_2_ diffusivities, this equilibration occurs essentially instantaneously. In the previous section, we exploited this fact to allow the determination of the concentration profiles for both components from the primary IFM data.

Already in the very first CO_2_ concentration profiles, we recognize the same feature as was seen in Fig. 3b for the profiles of ethane in a mixture with propene towards the end of our observations; concentrations close to the crystal boundary are significantly exceeded by those approaching the crystal centre. Once again, we note that the molecules (this time CO_2_) must have been able to diffuse ‘uphill' since otherwise intracrystalline concentrations exceeding those at the boundary would not be possible. Following the example given by Fig. 3, where uphill diffusion of ethane is driven by the decay in propene concentration towards the interior of the crystal, we now easily identify the decrease in the ethane concentration as the ‘driving force' for the uphill diffusion of CO_2_. We note that CO_2_ diffuses so rapidly that the uphill diffusion process has already been accomplished before we were able to record the first profiles.

Considering ethane as the ‘slow' molecule, we are now able to follow the whole process of equilibration (Fig. 2a). The propagation of the ethane diffusion front is accompanied by a continuous decrease of its slope. This leads, with equation (3), to a further decrease of the driving force which, at the beginning of the uptake experiment (and before our first measurement), was strong enough to direct a CO_2_ flux ‘uphill' into the crystal interior. Already with the very first profile shown in Fig. 2b, we have got beyond the range covered by the profiles shown in Fig. 3b. The progressive decay in the CO_2_ concentration indicates that, right from the beginning of our measurements, the CO_2_ fluxes are directed towards the crystal surface, that is, in the downhill direction for CO_2_. In our mixture uptake experiments with ethane and CO_2_, we are thus able to exactly follow this part of the history of overshooting that was inaccessible with mixtures of propene and ethane, that is, the approach to final equilibrium which, after overshooting, is controlled by the equilibration of the slower component.

### First-order predictions

Over the last few decades, benefitting from gains in the accuracy of experimental diffusion measurements, the prediction of diffusivities in zeolitic host–guest systems by molecular dynamics simulations^44^,^45^,^46^,^47^,^48^ has made impressive progress. Such simulations, involving solutions of the Newtonian equation under the force field arising from molecule–wall and molecule–molecule interactions, are often performed close to the limit of the available computational facilities. These complications may be avoided if the diffusion path of the molecules passes through narrow windows in which the accommodation probability of guest molecules is negligibly small in comparison with the cavities connected through these windows since, for such systems, the so-called ‘infrequent event techniques' (see, for example, chapter 9 in ref. 40) may be applied. Following the framework provided by classical transition state theory^49^,^50^,^51^, such predictions require only the information provided by the binary adsorption equilibrium isotherm for the host–guest system. First-order predictions of this type are especially useful for binary and multi-component systems, for which rigorous molecular dynamics simulations are complicated by the presence of more than one diffusing species.

The approach provided by transition state theory may be rationalized by exploiting the condition of dynamic equilibrium between the populations in the zeolite cavities and in the windows. Since the passage of a molecule through the window is considered as an ‘infrequent' event, any mutual interaction of molecules within the windows may be neglected. As a consequence, the mean transit time through a window is unaffected by the guest concentration and the relative window population increases in proportion to the pressure of the considered species in the surrounding atmosphere. The mean molecular lifetime within the individual cavities is thus immediately seen to be proportional to the ratio between the actual concentration and the partial pressure of the species under consideration. In this approach, a key quantity controlling the rate of molecular propagation is thus directly related to the equilibrium adsorption properties of the system. In the Methods section, we show that this correlation may be represented by the relations


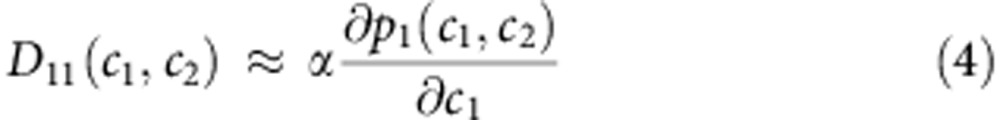


and


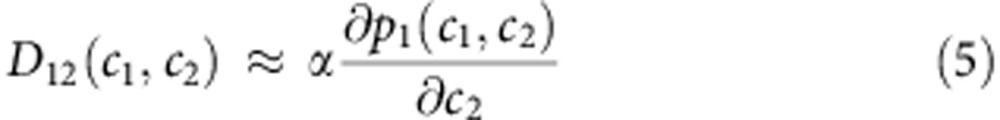


where the elements of the diffusion matrix are seen to be related to the partial derivatives of the partial pressures with respect to the local concentrations by a common factor (*α*) that is independent of the guest concentrations *c*_1_ and *c*_2_, and is proportional to the single-component diffusivity at zero loading.

**Figure 4:**
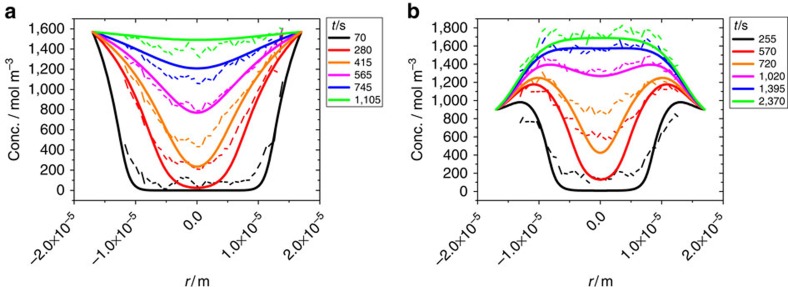
Profile evolution during two-component uptake estimated from the two-component isotherms. (**a**) Ethane (the ‘slow component') during two-component uptake in a mixture with CO_2_ (situation of Fig. 2), **b** shows ethane (the ‘fast component') during ethane uptake after propene presorption over 7 h (situation of Fig. 3). Thick lines are numerical solutions of Fick's second law with the concentration-dependent diffusivities as predicted by equations (4) and (5). Dashed lines show the experimental data of Fig. 2a) and Fig. 3b).

Figure 4 shows the transient concentration profiles of ethane resulting from equations (4) and (5) as the solutions of the diffusion equation under the initial and boundary conditions considered in our experiments (see Methods). The common factor *α* (determining the timescale) is the only free parameter in these calculations. For comparison, the corresponding experimental data are also indicated. The calculations using equations (4) and (5) are seen to reproduce all the main features as experimentally observed. In particular, the overshooting behaviour shown in Fig. 4b is correctly predicted. We also note that the most severe differences between the calculations and the experimental findings are observed towards the central axis of the crystals. This, however, is the behaviour to be expected based on the previous (single-component) uptake and release experiments with this type of crystal. Ideal ZSM-58 crystals allow diffusion only in the radial direction (see Fig. 1). However, uptake experiments with light hydrocarbons showed that, in real crystals, there must be a small flux in the axial direction since the concentration at the centre starts to increase before the diffusion fronts coming from the side faces have reached the centre^52^,^53^. This effect is not considered in our model, which is based on the assumption that the crystals have the ideal structure.

## Discussion

The application of IFM, which allows the measurement of transient concentration profiles in nanoporous crystals with submicron spatial resolution, has hitherto been restricted to single-component systems since, in general, the profiles for the individual components of a mixed adsorbed phase cannot be resolved. However, by considering the adsorption of mixtures of two components with very different intracrystalline diffusivities (CO_2_ and ethane, and ethane and propene, both in zeolite DDR), we succeeded in overcoming this limitation, thus making it possible to record the individual transient concentration profiles in a binary system with unprecedented spatial resolution. This new approach was applied to study two features of the ‘overshooting' phenomenon in which guest concentrations exceed their equilibrium values. First, it was shown that, in the presence of a non-uniform concentration of an essentially immobile component (propene) with its concentration decreasing towards the crystal interior, a second (faster) component (ethane) may diffuse ‘uphill' (that is, in the direction of increasing ethane concentration). To the best of our knowledge, this represents the first direct experimental demonstration of ‘uphill diffusion' (and the resultant ‘overshooting' behaviour) in a nanoporous adsorbent. A second set of experiments for the mixture ethane–CO_2_, in which ethane is now the slow component, allowed the attenuation and final disappearance of the overshoot to be studied in detail.

In addition to the advantages for IFM studies arising from the symmetry of the pore system, the windows of zeolite DDR are small enough to allow a straightforward first-order prediction of the relevant diffusivities as a function of the guest composition from transition state theory. The predicted transient concentration profiles show good agreement with the experimental data, thus confirming that the diffusional behaviour of the binary system can be adequately predicted from the binary equilibrium isotherm and the (single-component) diffusivities at zero loading, without recourse to detailed molecular simulations.

In addition to the relevance of these results for fundamental research, the systems studied are also of particular technological interest due to the potential application of DDR as a kinetically selective adsorbent for removal of CO_2_ from natural gas and for separation of light hydrocarbons.

## Methods

### Microimaging by IFM

IFM makes it possible to record transient concentration profiles of guest molecules in nanoporous host systems with a high spatial and temporal resolution. It is based on the principle that the optical density of light passing through a transparent porous crystal depends on the nature and amount of guest molecules present in its pore system. The primary information recorded by IFM measurements is the change in phase shift Δ(Δ*ϕ*) between a light beam passing through the sample and a reference beam passing through the surrounding gas phase. By using an interferometer of Mach–Zehnder type with shearing mechanism and phase shifter, both light beams are superimposed and an interference pattern is generated. A change in the phase shift is caused by a change in the refractive index of the host material Δ*n* when guest molecules are ad- or desorbed, leading to a change of this interference pattern. To a good approximation, the change in the phase shift Δ(Δ*ϕ*) is proportional to the change in the concentration of the guest molecules Δ*c*:


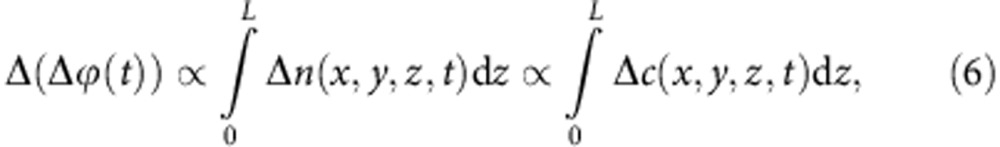


with *L* denoting the crystal height in axial direction. In IR reflectance measurements, changes in the refractive index (Δ*n*) can be used to measure thickness, roughness, guest loading and other characteristics of films and membranes of nanoporous materials as demonstrated by Nair and Tsapatsis^54^.

The measurements were carried out using an interference microscope of the type ‘Jenapol' (Carl Zeiss AG). Images were recorded by a CCD camera (SenSys KAF 0400, Photometrics), which is, together with the phase shifter of the microscope, controlled by a personal computer. Monochromatic light at a wavelength of 589 nm is used in our microscope. Furthermore, the set-up includes a static vacuum system consisting of vacuum pump, reservoir tank for guest molecules, several pressure gauges and sensors for adjusting the pressure to the required level. A few dozen DDR crystallites under study are placed in an optical cell, which is connected to the vacuum system.

Uptake or release experiments are initiated by step changes in the gas phase surrounding the crystals. IFM offers the potential to record maps of the intracrystalline distribution of guest molecules in large nanoporous crystals (10–500 μm) with a spatial resolution down to the pixel size of the CCD camera (0.45 μm × 0.45 μm) and a time resolution of about 10 s. The crystal under study should be transparent and exhibit nearly parallel and plane surfaces at top and bottom. Further details of the IFM technique may be found in literature^14^,^40^.

For mixtures of guest molecules, the change in the recorded signal contains contributions from both components which cannot be measured separately. However, the concentration profiles of both components can be easily calculated if the mobility of both components differs substantially. This is in particular true if the pore size is in the range of the critical diameter of the guest molecules—a condition that is usually found in technical applications based on the molecular sieving effect. Then, small differences in the guest size lead to dramatic differences in the diffusivities of both components. This opens the field for detailed investigations of various guest mixtures in different host systems of practical relevance.

### Transient uptake experiments

The DDR zeolites of the batch under study were very uniform in size. The crystallites had a diameter of 34 μm, the height was estimated to be about 20 μm. For further details of the synthesis, we refer to literature^22^,^53^.

The crystals were activated at 200 °C (heating rate of 1 K min^−1^) for about 10 h under vacuum. Subsequently, the sample was cooled down to room temperature (23 °C) and the cell was fixed under the microscope. All measurements presented here were carried out at this temperature. In the case of the ethane/CO_2_ experiment (see Fig. 2), a mixture of 200 mbar ethane and 200 mbar CO_2_ was established within the reservoir tank. To study the overshoot phenomenon, the activated crystals were then exposed to the gas mixture by a rapid change in the gas phase pressure (from vacuum to 400 mbar).

The sequence for the propene/ethane experiment was as follows: subsequent to a propene presorption for about 7 h, with a pressure of 10 mbar, the surrounding propene atmosphere was quickly removed (without changing the distribution of the propene molecules within the crystal). Then, the sample was immediately exposed to an atmosphere of 200 mbar ethane.

### Single-component profiles during ethane/CO_2_ uptake

Supplementary Fig. 1 shows the evolution of the phase shift Δ(Δ*ϕ*) as the primary experimental data provided by IFM on recording the uptake of ethane–CO_2_ mixtures on a crystal of zeolite ZSM-58. Equation (2), correlating the phase shift with the changes in concentration of the individual components, becomes


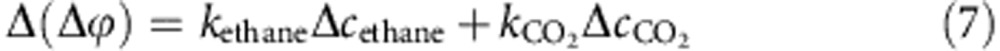


The factors *k*_ethane(CO_2_)_ relating the phase shifts to the concentration changes may be determined by comparing the conventionally measured (absolute) isotherms with the relative isotherms from the IFM signals for the pure components. Supplementary Fig. 2 shows the results of such studies in which we have compared our IFM measurements with literature data for the adsorption isotherms of the two guest molecules under study in zeolite DDR^25^,^55^. Differences in the heights *L* of the crystals must be accounted for. With the crystals used in this study, however, this precaution turned out to be unnecessary as the crystal height was very uniform. The almost-perfect agreement between the shape of the macroscopically determined adsorption isotherms (left ordinate scale in Supplementary Fig. 2a,b) with the phase shift equilibrium data obtained in our IFM microimaging experiments (right ordinate scale in Supplementary Fig. 2a,b) nicely confirms the validity of the assumption of proportionality between phase shift and changes in concentration for both of the considered molecules. This is illustrated in Supplementary Fig. 2c where the relation between the changes in phase shift and concentration appear as straight lines for each component, yielding *k*_ethane_=8.08 rad/(mol kg^−1^) and 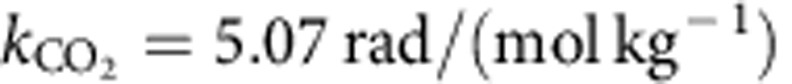 (see Supplementary Note 1 for data fitting).

Knowledge of the factors *k*_*i*_ in equations (2) or (7), 8 alone does not provide sufficient information to allow the changes in concentration Δ*c*_*i*_ of either component to be calculated from the measured changes Δ(Δ*ϕ*) in the phase of the light beam passing through the crystal under study. The required second equation is provided by the assumption of local equilibrium for the fast diffusing component (CO_2_). At equilibrium, the guest concentration of each component is determined by the pressures *p*_*i*_ of both components in the gas phase (*c*_*i*_=*c*_*i*_[*p*_*i*_,*p*_*j*_]). Equivalently, the mutual dependence of the two concentrations and their gas pressures at equilibrium may be expressed in the form *c*_*i*_=*c*_*i*_[*p*_*i*_, *c*_*j*_(*p*_*i*_, *p*_*j*_)] where now the concentration of component *j* is understood as the second independent variable which, together with the pressure *p*_*i*_ of the other component (which is assumed to be equilibrated over the sample), determines the concentration of component *j*. Our assumption of local equilibrium of CO_2_ throughout the crystal makes this choice of the notation more appropriate for our further discussion in which we consider the concentration of CO_2_ as a function of the local ethane concentration and the externally applied pressure of CO_2_:


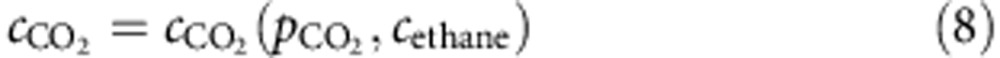


With the value of the CO_2_ pressure known for a given sorption experiment, by equation (8), the local concentration of CO_2_ is seen to be a function of only the local ethane concentration. Hence, with the functional relationship between the local concentrations of ethane and carbon dioxide, shown to exist by virtue of equation (8), we have sufficient information to deduce (from equation (7)) the concentration profiles of both components from the profiles of the overall phase shifts.

IAST^35^ has proved to be the most universally applicable approach for predicting the equilibrium concentrations in two-component adsorption from the single-component adsorption isotherms for both components. In detailed investigations employing Grand Canonical Monte Carlo simulations for mixture adsorption in DDR^36^, IAST has been found to serve as a good approach in the range of small and modest gas phase pressures as considered in this study. Supplementary Fig. 3 shows the relevant functional dependency 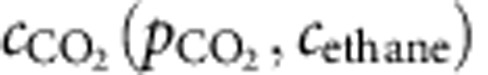 as implied by equation (8), which has been calculated by means of the IAST from the single-component adsorption isotherms displayed in Supplementary Fig. 2a,b for the experimental condition relevant in our experiments, that is, for a partial gas phase pressure 
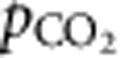
 of about 200 mbar of carbon dioxide and local concentrations *c*_ethane_ varying from 0 up to about 1 mol kg^−1^ (corresponding to the interval of concentrations covered by single-component uptake for a pressure step from zero to about 200 mbar ethane pressure). The relation between the two concentrations is thus seen to yield a simple linear dependency


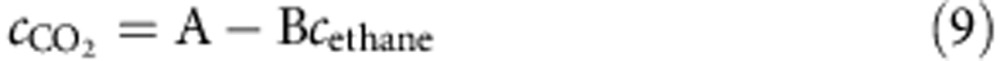


By inserting the respective expression for 
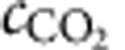
 or *c*_ethane_ provided by equation (9) into equation (7), the individual intracrystalline concentration for each component is obtained as presented in the Fig. 2a,b.

### Single-component profiles of ethane with presorbed propene

Conversion of the phase shifts into absolute single-component concentration values was based on the adsorption isotherms of Hedin *et al*.^55^ as shown in Supplementary Fig. 4. Determination of the total propene loading did, moreover, necessitate the performance of a whole cycle of ad- and desorption processes as shown in Supplementary Fig. 5. Total propene loading results as the very last step in the series of experiments since reactivation of the crystal under study is, under the given experimental conditions, essentially impossible within a reasonable timescale. It yields a conversion factor of *k*_propene_=10.67 rad/(mol kg^−1^) (or *k*_propene_=6.06 × 10^−3^ rad/(mol m^−3^), with a crystal density 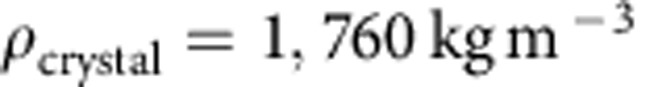).

Since, close to the boundary, the crystals are not transparent, the recorded profiles do not extend right to the crystal surface. The propene concentration directly at the crystal boundaries is assumed to correspond to the total propene loading (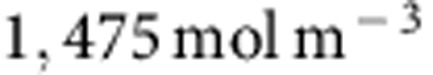 or 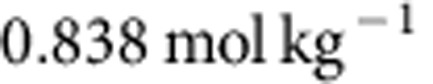), since no dominating surface barrier is expected.

The maximum ethane concentration for this crystal has been estimated in the following way: in the centre part of the propene profile after presorption (see Fig. 3a), the propene concentration was found to be ∼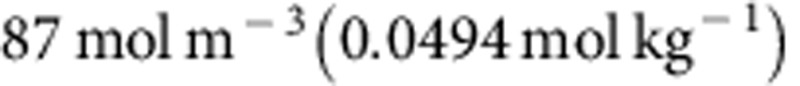. On the basis of this information, the local ethane concentration for *p*_ethane_=200 mbar can be calculated directly by IAST. Thus, the ethane concentration in the centre region of the saturation profile was found to be about 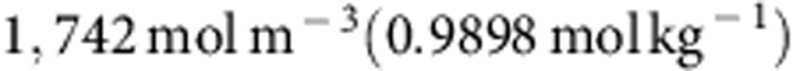. This results in a conversion factor of *k*_ethane_=4.22 × 10^−3^ rad/(mol m^−3^) (or *k*_ethane_=7.42 rad/(mol kg^−1^)).

Furthermore, the ethane concentration at the crystal surface had to be estimated, since this parameter plays an important role as one of the boundary conditions for solving Fick's second law of diffusion. Applying IAST for a propene concentration of 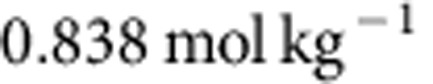 (which is the propene concentration at the surface) and *p*_ethane_=200 mbar, an ethane concentration of 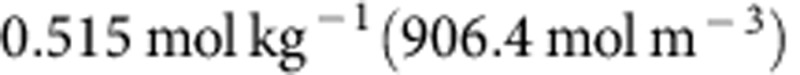 is found, which is denoted as *c*_10_. This concentration of ethane is maintained at the crystal border (at *r*=*R*) for all times *t*>0 (resulting in a coincidence of all profiles at this point), where we imply that there are no barriers. With these considerations, we arrive at the concentration profiles in absolute concentration values as presented in Fig. 3a,b.

### Diffusion equation

We have to solve Fick's second law for two-component diffusion


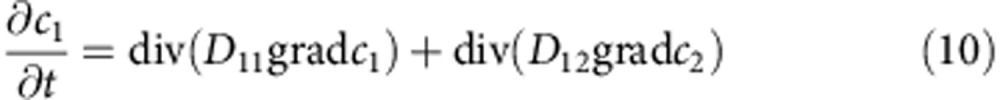


in cylindrical symmetry


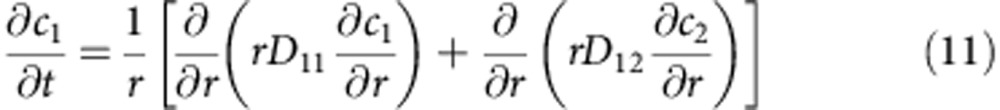


For a solution, the equation must be regularized and appropriate initial and boundary conditions need to be defined. For both experiments presented in the manuscript, the problem that needs to be solved for achieving the evolution of the ethane concentration with time, *c*_1_(*r*, *t*), is formulated as follows:

Let *ɛ*>0 be small and *R*<*R*_0_ close to *R*. The problem is to find *c*_1_(*r*, *t*) on (0, *R*) × (0, ∞) of


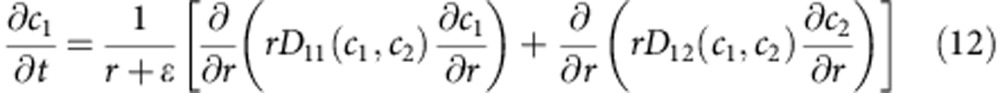


with


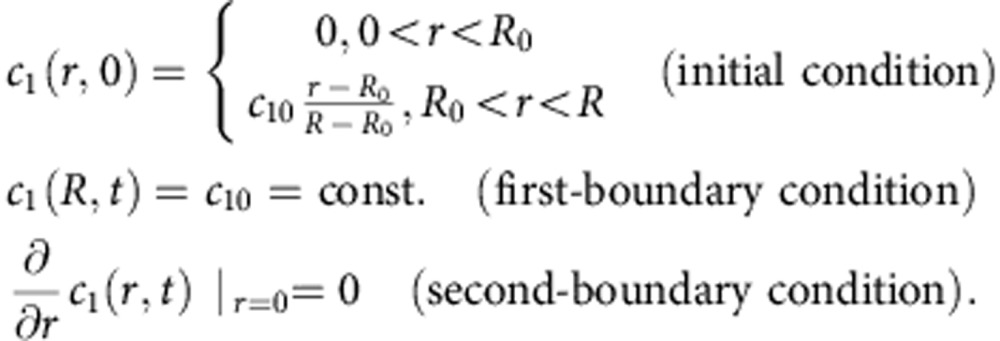


A numerical solution for *c*_1_(*r*, *t*) according to equation (12) can be achieved for two special cases: first, if *c*_2_ is independent of time, it can easily be replaced by a fit curve depending on only the radius *r*, as it would be the case for propene. Second, if *c*_2_ can directly be expressed by a function of *c*_1_(*r*, *t*). This is the case for CO_2_, which is assumed to be instantaneously equilibrated. Furthermore, eligible models for *D*_11_(*c*_1_, *c*_2_) and *D*_12_(*c*_1_, *c*_2_) are needed. These models will be provided by analysis of the data maintained by the approaches made for *D*_11_ and *D*_12_ (Supplementary Figs 6 and 7).

For solving equation (12) numerically by a computational software, a replacement of the initial condition given above turned out to be necessary in practice, due to the incidence of an inconsistency problem at *r*=*R*_0_. This problem was successfully overcome by expressing the initial condition as a Heaviside function, which is zero for all *r* except from *r*=*R*_0_. At *R*_0_, *c*_1_(*R*_0_, 0)=*c*_10_ is valid.

### First-order prediction of diffusivities

We exploit the special geometry of the pore structure of ZSM-58 (see Fig. 1c) to derive simple relations for *D*_11_(*c*_1_, *c*_2_) and *D*_12_(*c*_1_,*c*_2_) from transition state theory. Given the existence of narrow windows between the individual cages, the diffusion of guest molecules in the ZSM-58 pore system may be described by a simple jump model^40^,^50^.

The total number of molecules jumping, per unit time, between two adjacent cages of separation *λ* from, say, left to right is given by:


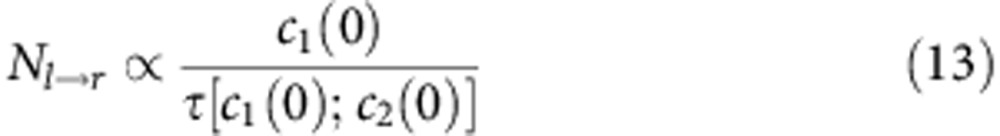


and, correspondingly, by


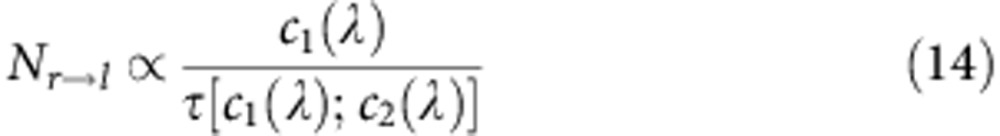


for molecules jumping from right to left.

The mean molecular lifetime, denoted as *τ*(*c*), within the individual cavities is proportional to the ratio between the actual concentration and the partial pressure of the species. Thus, one may note


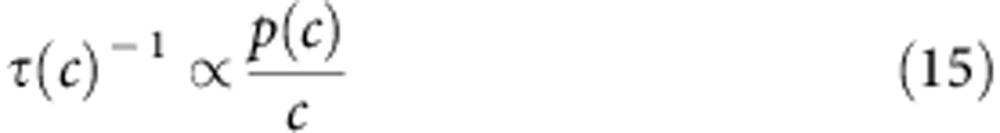


or, in more detail:


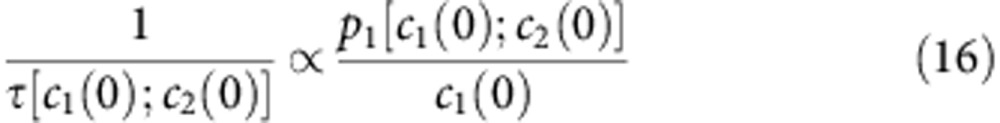


and


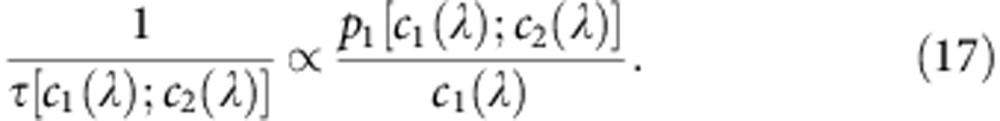


Replacing the ‘*τ*-terms' in equations (13) and (14) by the thus resulting expressions yields


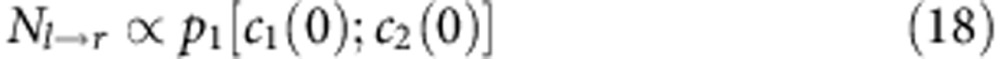


and


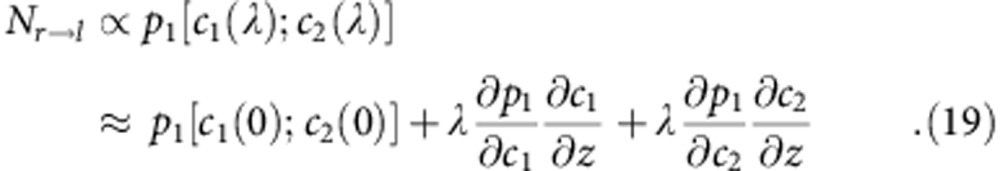


Combing the last two relations yields


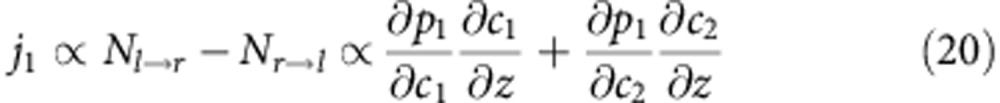


where we have taken account of the proportionality between the net number of jumps and the flux into a given direction.

Comparison with Fick's first law of diffusion


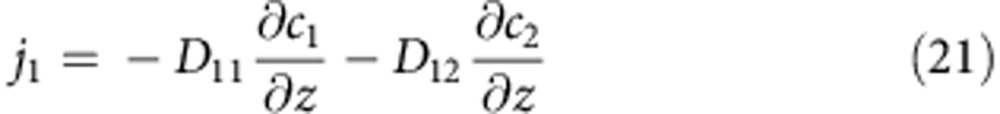


results, finally, in the expressions


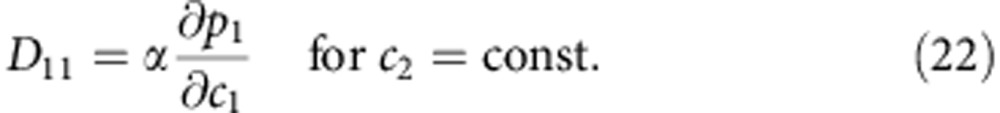


and


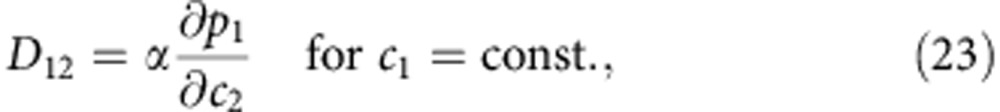


with *α* denoting a factor of proportionality, which is seen to be equal to the single-component diffusivity at zero loading divided by the Henry constant.

One may immediately arrive at these relations by inserting the expression of the self-diffusivity as derived by transition state theory (Equation (4.42) in ref. 40) into Equation (3.37) of ref. 40, which correlates the elements of the diffusion matrix with the self-diffusivities for negligible cross-correlation, that is, for vanishing phenomenological cross-coefficients (*L*_*ij*_=0 for *i*≠*j*) correlating the fluxes with the gradients of their chemical potentials of different molecular species. Such cross-correlations may in fact be neglected, given the low occupation probability of ‘window' positions as assumed in our simplified model.

### Concentration dependence of the diffusivities

To determine the partial derivatives appearing in equations (22) and (23), we have to know the dependence *p*_1_(*c*_1_, *c*_2_) of the pressure of the relevant diffusant on the concentrations of both components under equilibrium. In co-adsorption with ethane, CO_2_ may be assumed to be instantaneously equilibrated throughout the crystal. The CO_2_ concentration at any position in the crystal is therefore determined by the local ethane concentration and the fixed value of *p*_2_=200 mbar for the CO_2_ pressure. Correspondingly, also the equilibrium ethane pressure *p*_1_ at any position corresponding to the local concentrations of the two components is fixed and determined by these concentrations. IAST was applied for either constant *c*_2_ and a fine-meshed list of *p*_1_ values, or for constant *c*_1_ and a fine-meshed list of *p*_2_ values. Subsequently, numerical differentiation of the respective lists was performed to achieve the list of values for 
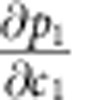
 and 
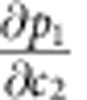
. The, thus, calculated values of 
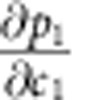
 and 
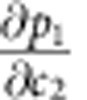
 are found to reveal a linear dependence as shown in Supplementary Fig. 6a. Supplementary Fig. 6b represents the dependence of 
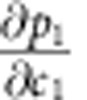
 and 
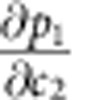
 on the concentrations *c*_1_ and *c*_2_. Supplementary Fig. 6c,d show the surface fit of the approach


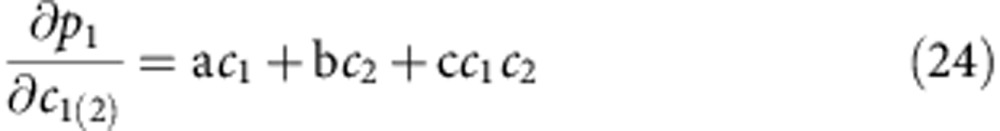


used in the numerical solution.

Supplementary Fig. 7a,b shows the partial derivatives of the (equilibrium) ethane pressure as a function of the ethane concentration (varying during the course of the experiment) and the propene concentration (essentially remaining constant) as determined from the IAST predictions of mixture adsorption. The numerical solution was based on the approach


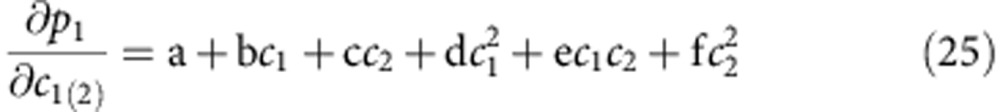


as illustrated by Supplementary Fig. 7c,d.

### Diffusive fluxes and zero-loading diffusivities

By recording the transient concentration profiles during molecular uptake and release, microimaging also provides all information necessary for the measurement of the intracrystalline diffusive fluxes. Thus, for the system under study, molecular fluxes entering the individual crystal in a time interval *t*_1_…*t*_2_ are easily seen to be correlated with the evolution of the intracrystalline concentrations by the relation


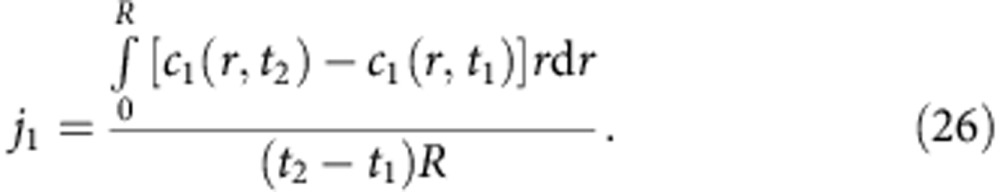


For a proper calculation of the integral, the measured data were fitted by either fifth-order polynomials or asymmetric double sigmoidal functions. The resulting fluxes have to be attributed to mean ethane concentrations (concentrations between two adjacent profiles). Supplementary Fig. 8a shows the ethane profiles thus analysed during co-adsorption with CO_2_, with the fluxes resulting via equation (26) in Supplementary Fig. 8b. Supplementary Fig. 8c compares the thus determined fluxes with the values resulting from Supplementary equations (1) and (2) (see Supplementary Note 2) with the concentration gradients taken from the transient profiles, the prefactors *D*_11_ and *D*_12_ as predicted with equations (22) and (23) and with *α*=4.8 × 10^−16^. Supplementary Fig. 9a–c show the corresponding plots observed during ethane uptake with pre-adsorbed propene. Here best fit is attained with a value of *α*=4 × 10^−16^ (Supplementary Note 2). This similarity in the best fit values has to be implied since, with equation (22), *α* is seen to be the diffusivity at zero loading divided by the Henry constant.

## Additional information

**How to cite this article:** Lauerer, A. *et al*. Uphill diffusion and overshooting in the adsorption of binary mixtures in nanoporous solids. *Nat. Commun.* 6:7697 doi: 10.1038/ncomms8697 (2015).

## Supplementary Material

Supplementary Information Supplementary Figures 1-9, Supplementary Notes 1-2 and Supplementary References
