# Supplementary material for: Uphill diffusion and overshooting in the adsorption of binary mixtures in nanoporous solids
Source: Nat Commun. 2015 Jul 16;6:7697. doi: 10.1038/ncomms8697 (PMC4518250; doi:10.1038/ncomms8697)
Supplement: Supplementary Information — Supplementary Figures 1-9, Supplementary Notes 1-2 and Supplementary References [file ncomms8697-s1.pdf]

Supplementary Figure 1

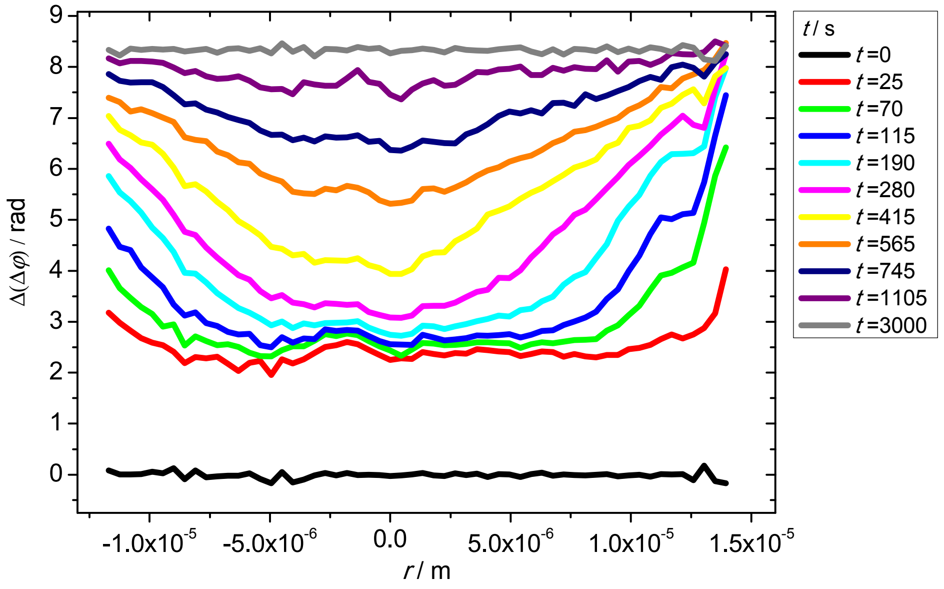

Supplementary Figure 1 Evolution of the IFM phase shift after exposing an initially empty crystal, essentially instantaneously, to a surrounding atmosphere of ethane and CO_2 with partial pressures each of 200 mbar.

Supplementary Figure 2

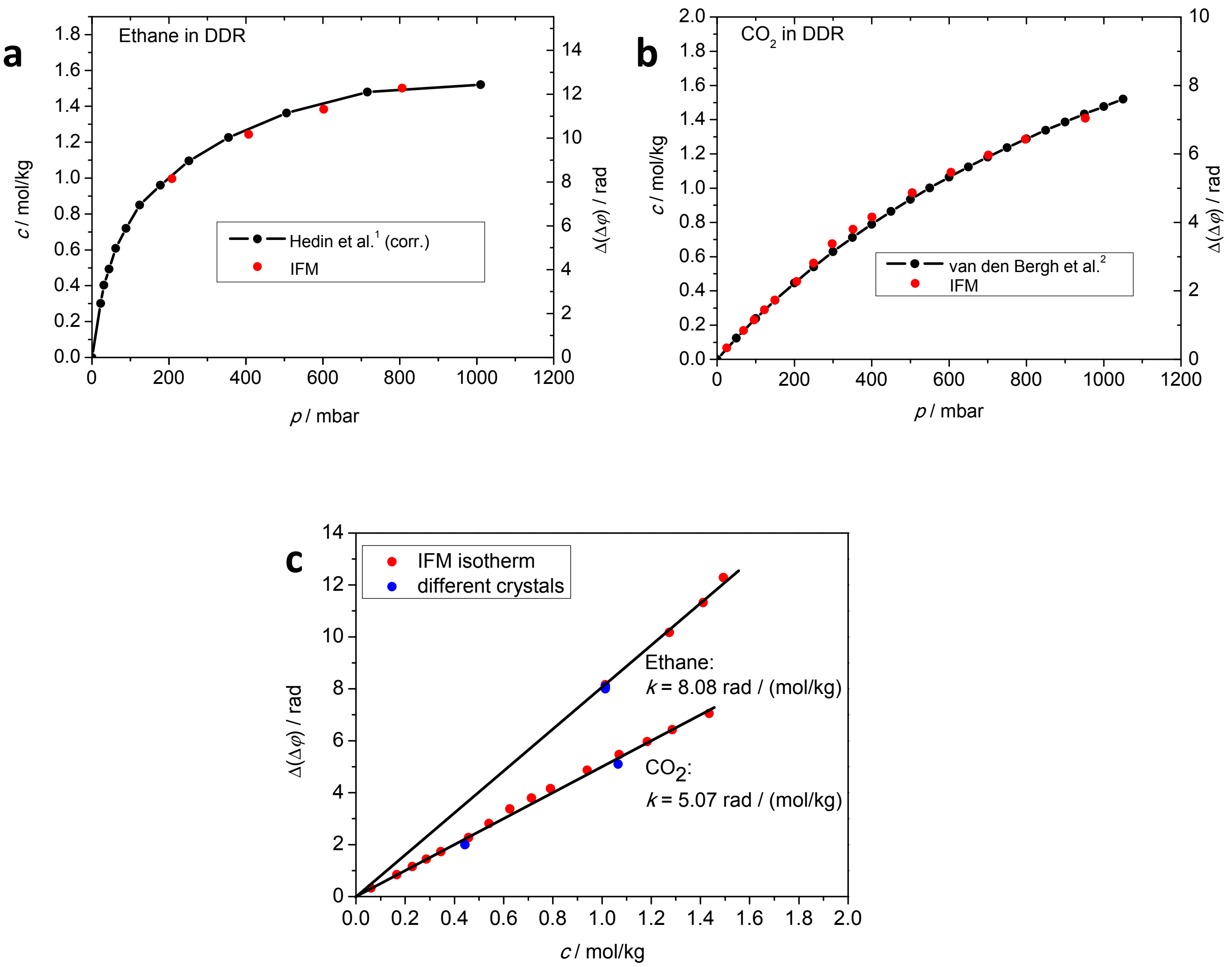

Supplementary Figure 2 Comparison of adsorption isotherms from interference microscopy (phase shift, right ordinate) and literature (macroscopic uptake from ref. 1,2, left ordinate) for ethane **a)** and CO_2 **b)** in zeolite DDR at room temperature. The agreement between IFM single crystal isotherms and literature data allows the determination of k_i (shown in **c)**), i.e. the factor of proportionality between the intracrystalline concentration c and the IFM phase change $\Delta(\Delta\varphi)$. **c)** additionally shows data from different crystals demonstrating single crystal conformity.

Supplementary Figure 3

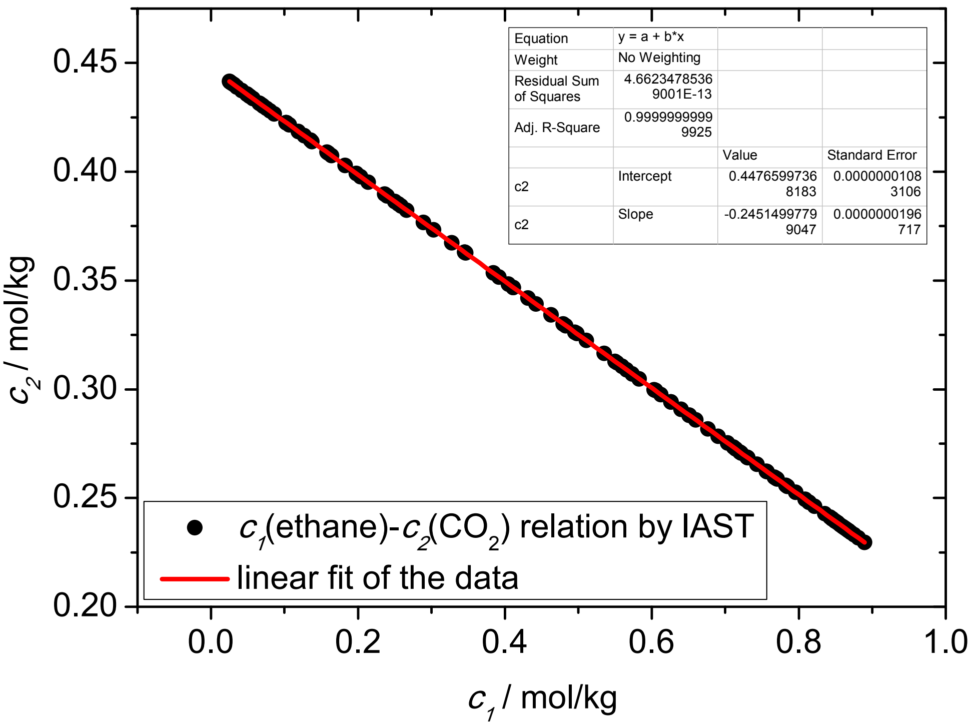

Supplementary Figure 3 IAST calculations for binary mixture adsorption with constant pressure of CO_2 of 200 mbar. The concentration of ethane varies from zero to the equilibrium concentration.

Supplementary Figure 4

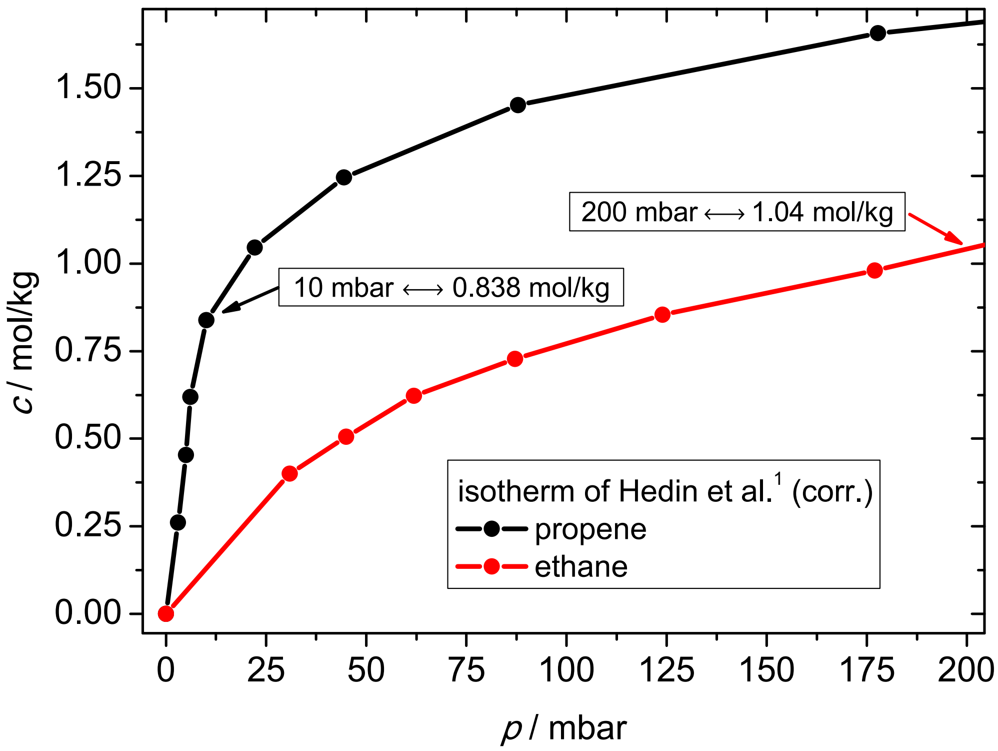

Supplementary Figure 4 Corrected isotherm (multiplied by a factor of 1.37, see Supplementary note 1) of Hedin et al.[1] for propene (black) and ethane (red). The loadings for 10 mbar propene and for 200 mbar ethane as used in the experiment are given separately in the boxes.

Supplementary Figure 5

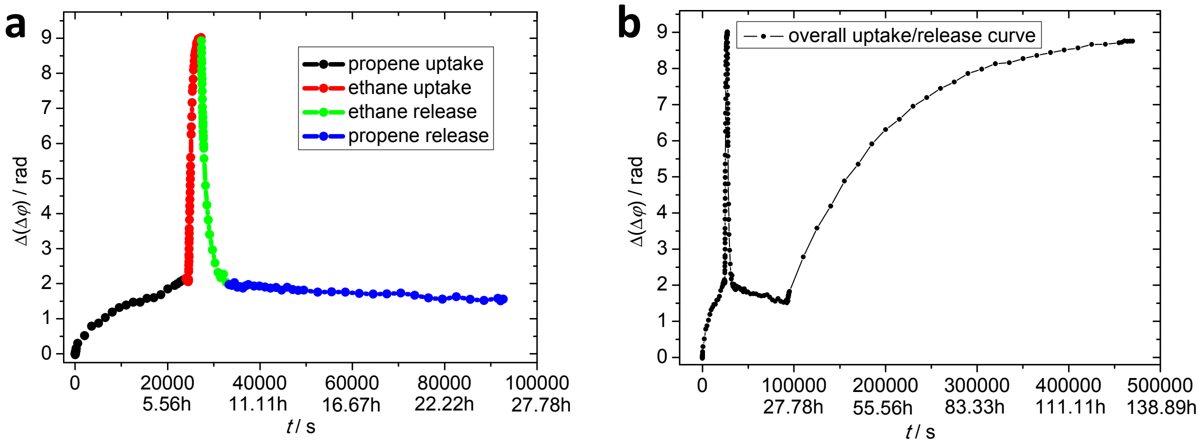

Supplementary Figure 5 In **a)**, the curves for propene and ethane uptake, as well as for their subsequent release are shown. The individual parts of the curve are colored differently. The overall uptake and release curve of the experiment is presented in **b)**. At about 92000 s, "long-term" adsorption at 10 mbar propene pressure is initiated.

Supplementary Figure 6

(1=ethane; 2=CO_2)

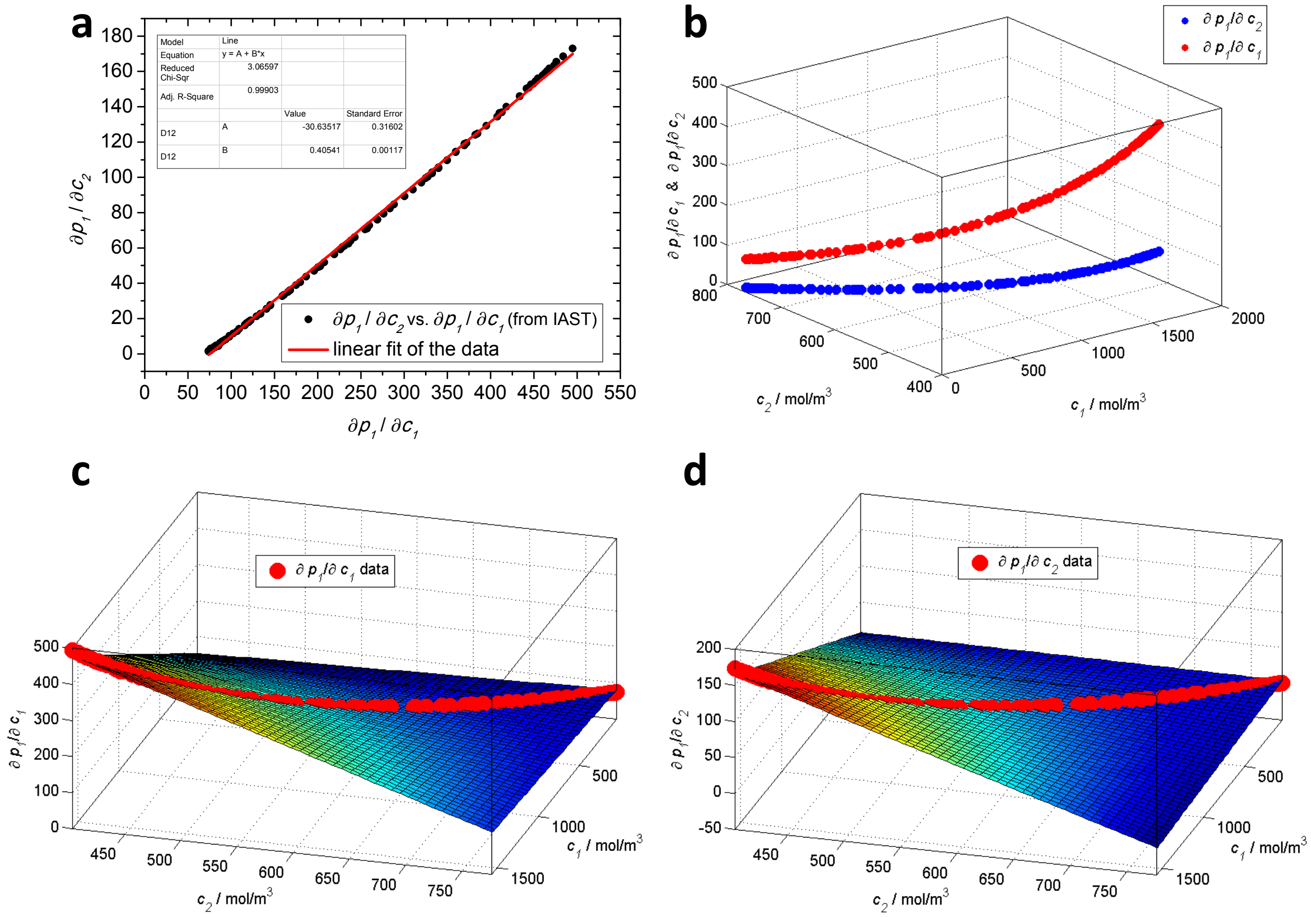

Supplementary Figure 6 a) shows the values of $\frac{\partial p_1}{\partial c_2}$ plotted versus $\frac{\partial p_1}{\partial c_1}$ as achieved by IAST calculations and subsequent numerical partial differentiation, with p_1 in mbar, c_i in mol/m^3. The values of $\frac{\partial p_1}{\partial c_2}$ (blue dots) and $\frac{\partial p_1}{\partial c_1}$ (red dots) plotted versus the concentrations c_1 and c_2 are presented in **b)**. Surface fit (by Eq. (24)) of the $\frac{\partial p_1}{\partial c_1}$ data is shown in **c)**, and of the $\frac{\partial p_1}{\partial c_2}$ data in **d)**, respectively.

Supplementary Figure 7

(1=ethane; 2=propene)

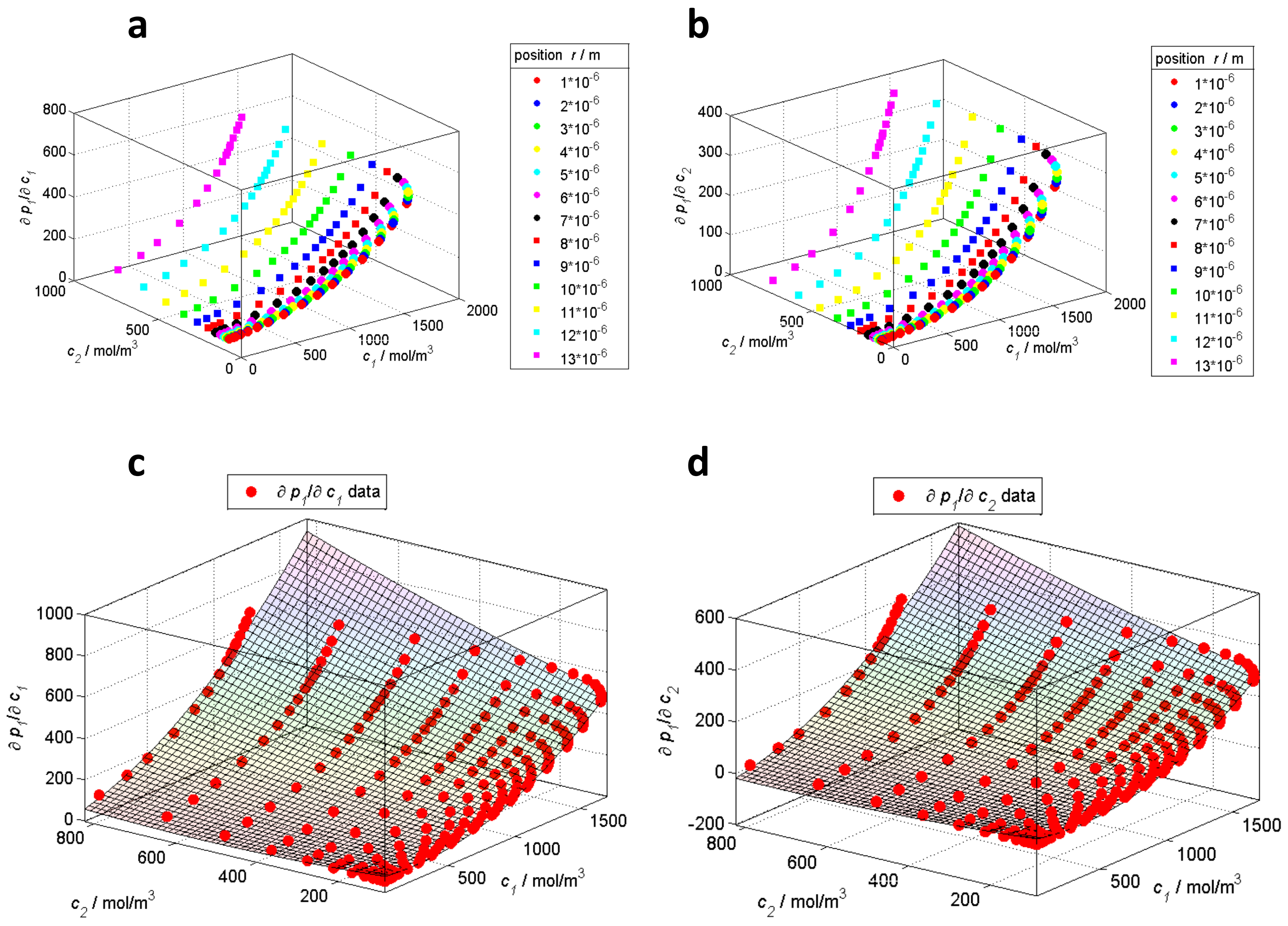

Supplementary Figure 7 The dependence of $\frac{\partial p_1}{\partial c_1}$ on the concentrations c_1 and c_2 is presented in **a)**, the dependence of $\frac{\partial p_1}{\partial c_2}$ in **b)**, respectively. Different symbols and colors are used for indicating the different locations and, hence, propene concentrations c_2, for which the derivatives have been calculated. The range of c_1 data corresponds with the range of ethane concentration covered in the experiment. Surface fits of the $\frac{\partial p_1}{\partial c_1}$ and the $\frac{\partial p_1}{\partial c_2}$ data by the approach provided by Eq. (25) are shown in **c)** and **d)**.

Supplementary Figure 8

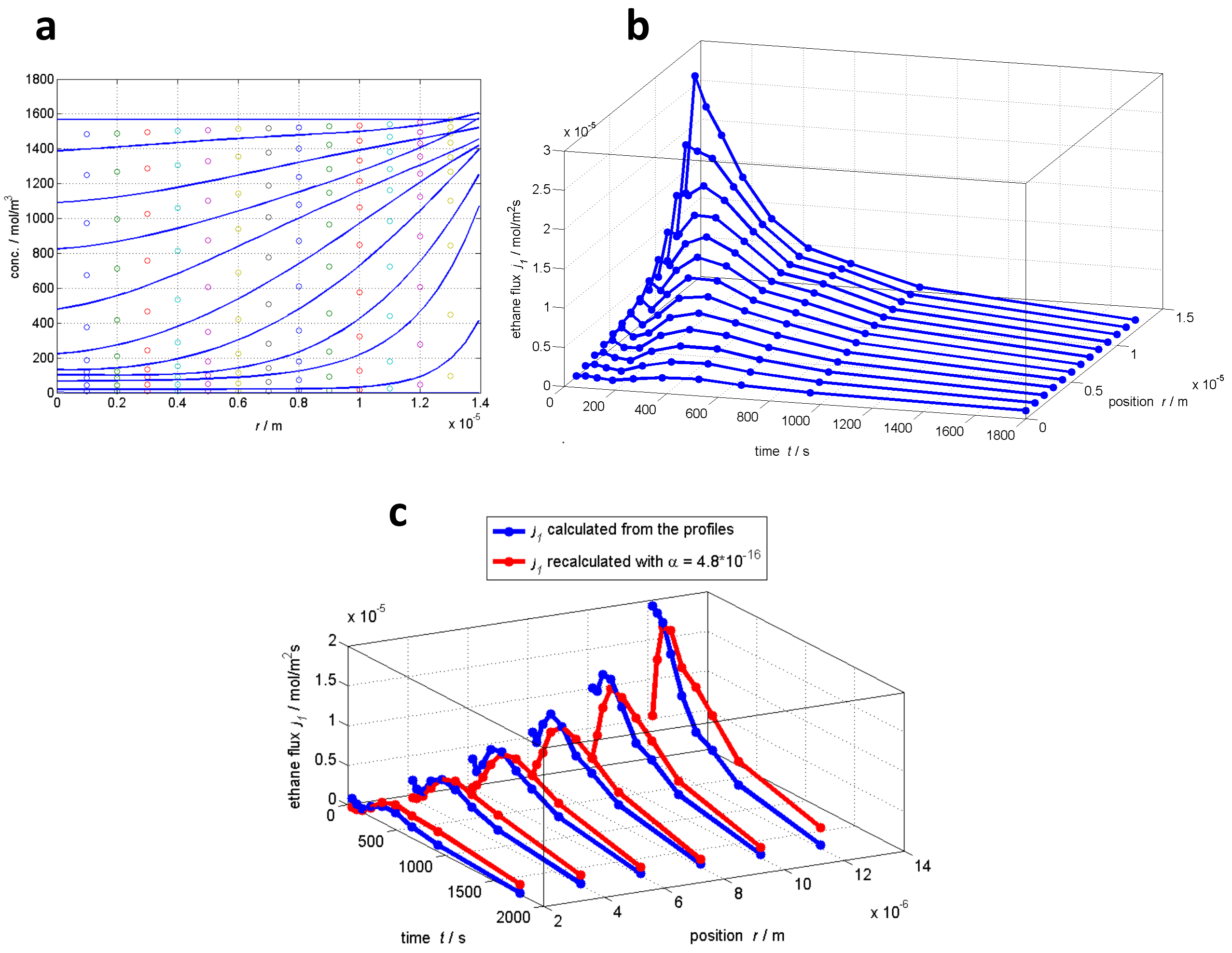

Supplementary Figure 8 a) shows the fit curves representing the left half of the ethane profiles (see Fig. 2a in the main paper), jointly with the mean ethane concentrations marked by colored circles. **b)** represents the ethane fluxes calculated according to Eq. (26) plotted against the time (mean time of two adjacent concentration profiles) and against the position r, respectively. A comparison of the fluxes determined from the evolution of the concentration profiles via Eq. (26) with those predicted via Fick´s generalized 1st law, Eq. (3), with the diffusivities resulting from the binary adsorption isotherms via Eqs. (4) and (5) with $\alpha = 4.8 \text{ x } 10^{-16}$ and the measured concentration gradients as specified by Supplementary Eqs. (1) and (2), is given in **c)**.

Supplementary Figure 9

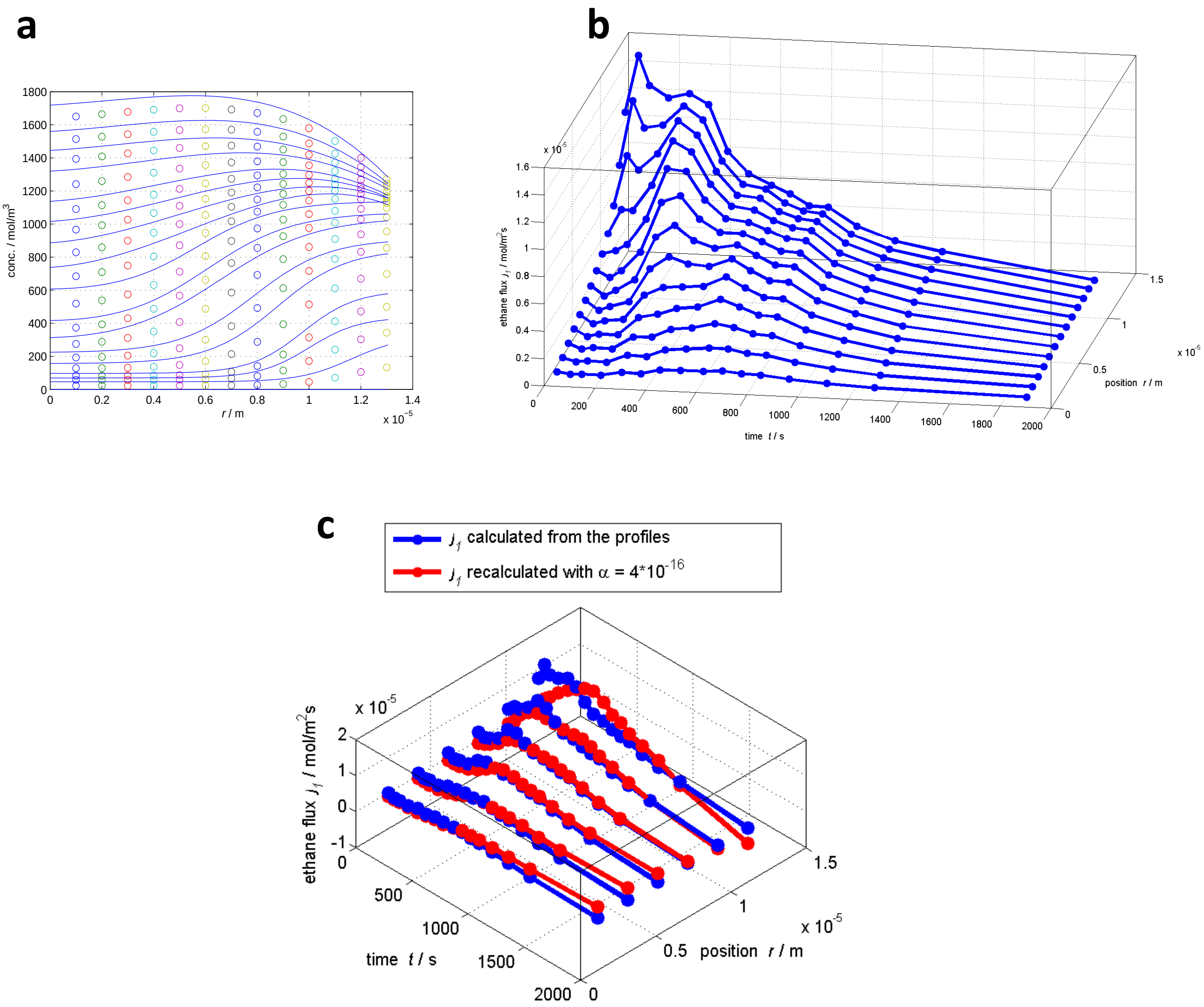

Supplementary Figure 9 a) shows the fit curves representing the right half of the ethane profiles (see Fig. 3b in the main paper), jointly with the mean ethane concentrations marked by colored circles. In **b)** the ethane fluxes calculated according to Eq. (26) plotted against the time (mean time of two adjacent concentration profiles) and against the position *r*, respectively, are shown. A comparison of the fluxes determined from the evolution of the concentration profiles via Eq. (26) with those predicted via Fick´s generalized 1st law, Eq. (3), with the diffusivities resulting from the binary adsorption isotherms via Eqs. (4) and (5) with $\alpha = 4 \text{ x } 10^{-16}$ and the measured concentration gradients as specified by Supplementary Eqs. (1) and (2), is given in **c)**.

Supplementary Note 1

Uncertainties in the determination of the k_i factors may arise from the fact that there are no data available in literature reporting single component isotherms for ethane and CO_2 simultaneously. While ref. 1 provides data for methane, ethane and other molecules for DDR zeolites with a Si/Al ratio of 190 at a temperature of 301 K, ref. 2 reports about CO_2, methane and other molecules for all-silica DDR crystals at 298 K. Due to the slightly different composition of the DDR zeolites and slightly different temperatures, the isotherms for methane from both publications differ. By treating the data of the methane isotherm of ref. 1 with a scaling factor of 1.37, good agreement with the methane isotherm of ref. 2 was achieved. This factor has also been applied to the ethane data of ref. 1 (and later on also to the propene data) so that this should represent the all-silica isotherms at room temperature. The modified ethane isotherm from ref. 1 and the unmodified CO_2 isotherm of ref. 2 were used for obtaining the dual-site-Langmuir parameters necessary for IAST calculations.

Supplementary Note 2

With the relevant elements of the diffusion matrix, given by Eqs. (4) and (5) of the main paper as a first-order estimate based on TST, Fick´s first law becomes

$$j_1 = -\alpha \frac{\partial p_1}{\partial c_1}\frac{\partial c_1}{\partial r} - \alpha \frac{\partial p_1}{\partial c_2}\frac{\partial c_2}{\partial r} = -\alpha(\mathrm{X}+\mathrm{Y}) \ . \qquad \text{(Supplementary Eq. 1)}$$

Written in vectors the ethane fluxes can be expressed by

$$\begin{pmatrix} j_{1,1} \\ \vdots \\ j_{1,n} \end{pmatrix} = \begin{pmatrix} \mathrm{X}_1 \, \mathrm{Y}_1 \\ \vdots \ \vdots \\ \mathrm{X}_\mathrm{n} \, \mathrm{Y}_\mathrm{n} \end{pmatrix} \begin{pmatrix} -\alpha \\ -\alpha \end{pmatrix} = \mathrm{M}x \ . \qquad \text{(Supplementary Eq. 2)}$$

In fact all quantities necessary for estimating α are known. Achieving of the set of fluxes was described in Methods, Eq. (26). The mean ethane gradients $\frac{\partial c_1}{\partial r}$ were calculated on the basis of the data shown in Supplementary Fig. 8a and 9a. Analogously the mean CO_2/propene gradients $\frac{\partial c_2}{\partial r}$ have been calculated by using a fitted version of concentration profiles shown in Figure 2b/3a in the main paper (mainly 5th order polynomials were used as fit functions). The procedure to obtain the partial derivatives $\frac{\partial p_1}{\partial c_1}$ and $\frac{\partial p_1}{\partial c_2}$ is also topic of the methods section. Thus we can solve the overdetermined system of equations given by Supplementary Eq. (2) for α. With the method of the Moore–Penrose pseudoinverse, α can be determined in such a way that it satisfies the set of equations as good as possible. The main principle behind this method can be described as follows: Instead of finding an x that solves the equation, one finds an x that minimizes $\|y\|_2{}^2$, where $y = \mathrm{M}x - j_1$ is the residual.

Using this method values of $\alpha = 4.8 \text{ x } 10^{-16}$ (for ethane/CO_2) and $\alpha = 4 \text{ x } 10^{-16}$ (for ethane/propene) were estimated. For a check-up, α was reinserted into Supplementary Eq. (2) for recalculating the fluxes (the same values for $\frac{\partial p_1}{\partial c_1}\frac{\partial c_1}{\partial r}$ and $\frac{\partial p_1}{\partial c_2}\frac{\partial c_2}{\partial r}$ have been used as for solving for α). The comparison of the raw data of the original fluxes and the recalculated ones is presented in the Supplementary Figs. 8c and 9c, showing satisfactory agreement with, most importantly, identical features with respect to both their time and space dependence.

Supplementary References

1. Hedin, N. DeMartin, G. J. Roth, W. J. Strohmaier, K. G. Reyes, S.C. PFG NMR self-diffusion of small hydrocarbons in high silica DDR, CHA and LTA structures, *Micro. Mesopor. Mater.* **109**, 327–334 (2008).

2. van den Bergh, J. Zhu, W. Gascon, J. Moulijn, J. A. Kapteijn, F. Separation and permeation characteristics of a DD3R zeolite membrane, *J. Membr. Sci.* **316**, 35–45 (2008).
